# Multiple Kinases Involved in the Nicotinic Modulation of Gamma Oscillations in the Rat Hippocampal CA3 Area

**DOI:** 10.3389/fncel.2017.00057

**Published:** 2017-03-06

**Authors:** JianGang Wang, XiaoLong He, Fangli Guo, XiangLin Cheng, Yali Wang, XiaoFang Wang, ZhiWei Feng, Martin Vreugdenhil, ChengBiao Lu

**Affiliations:** ^1^Key Laboratory for the Brain Research of Henan Province, Xinxiang Medical UniversityXinxinang, China; ^2^Department of Pathophysiology, Xinxiang Medical UniversityXinxinang, China; ^3^Key Laboratory of Neuronal Oscillation and Disease, Yantze University Medical SchoolJingZhou, China; ^4^Department of Neurobiology and Physiology, Xinxiang Medical UniversityXinxinang, China; ^5^Department of Laboratory Medicine, Yantze University Affiliated HospitalJingZhou, China; ^6^Department of Psychology, Xinxiang Medical UniversityXinxinang, China; ^7^School of Life Sciences, Birmingham City UniversityBirmingham, UK

**Keywords:** hippocampus, nAChR, PKA, PKC, ERK1/2, PI3 kinase, Akt, γ oscillations

## Abstract

Neuronal synchronization at gamma band frequency (20–80 Hz, γ oscillations) is closely associated with higher brain function, such as learning, memory and attention. Nicotinic acetylcholine receptors (nAChRs) are highly expressed in the hippocampus, and modulate hippocampal γ oscillations, but the intracellular mechanism underlying such modulation remains elusive. We explored multiple kinases by which nicotine can modulate γ oscillations induced by kainate in rat hippocampal area CA3 *in vitro*. We found that inhibitors of cyclic AMP dependent kinase (protein kinase A, PKA), protein kinase C (PKC), N-methyl-D-aspartate receptor (NMDA) receptors, Phosphoinositide 3-kinase (PI3K) and extracellular signal-related kinases (ERK), each individually could prevent the γ oscillation-enhancing effect of 1 μM nicotine, whereas none of them affected baseline γ oscillation strength. Inhibition of the serine/threonine kinase Akt increased baseline γ oscillations and partially blocked its nicotinic enhancement. We propose that the PKA-NMDAR-PI3K-ERK pathway modifies cellular properties required for the nicotinic enhancement of γ oscillations, dependent on a PKC-ERK mediated pathway. These signaling pathways provide clues for restoring γ oscillations in pathological conditions affecting cognition. The suppression of γ oscillations at 100 μM nicotine was only dependent on PKA-NMDAR activation and may be due to very high intracellular calcium levels.

## Introduction

Hippocampal oscillations reflect the coordinated, rhythmic activity emerging from mutually connected interneuron and principal cell populations. Fast network oscillations in the gamma frequency band (30–80 Hz, γ) are associated with higher brain functions, such as visual attention and memory (Fries et al., 2001; Lutzenberger et al., 2002; Jensen et al., 2007). γ oscillations provide the ms-precision timing matrix necessary for effective information processing and the maintenance of behaviorally relevant information in working memory (Roux et al., 2012). γ oscillations are impaired in neuropsychological disorders such as schizophrenia and Alzheimer’s disease which may explain the deficits of working memory in these disorders (Cho et al., 2006; Leicht et al., 2010; Klein et al., 2016).

Nicotine modulates neuronal network activity via nicotinic acetylcholine receptor (nAChR) activation. Nicotine induced hippocampal theta oscillations (Lu and Henderson, 2010) and increased the auditory-evoked or the stimulation-evoked hippocampal γ oscillations (Song et al., 2005; Featherstone et al., 2012), which may well explain the improvement of cognitive function in both normal subjects and patients with schizophrenia (Phillips et al., 2007). Recently, we found that nicotine at a low concentration increases hippocampal γ oscillations induced by kainate *in vitro*, while at a high concentration, nicotine decreases γ activity (Wang et al., 2015). However, the intracellular mechanism involved in nicotinic modulation of γ oscillation is unclear.

nAChR are ligand-gated cation selective channels that are widely distributed in the brain and classified as non-α7 and α7 nAChR subtypes, based on the calcium permeability of these channels, with α7 nAChR having relative high calcium permeability (Hu et al., 2002). These nAChRs may be selectively expressed in specific neuronal populations, and modulate neuronal function. For example, the selective expression of α2 nAChR in a subset of hippocampus area CA1 interneurons (Oriens-Lacunosum Moleculare cells) modulates information flow and long-term potentiation (LTP) in the CA1 region (Leão et al., 2012).

Nicotine activates cAMP-dependent protein kinase A (PKA) and extracellular signal-related kinase (ERK 1/2) via α7 nAChR activation in hippocampal neurons (Dajas-Bailador et al., 2002). The cAMP/PKA-mitogen-activated protein kinase (MAPK) pathway is a signal-transduction cascade which is neuron-specific (Waltereit and Weller, 2003). In addition, nicotine may also activate protein kinase C (PKC) to increase the density of non-α7 nAChR (α4β2 nAChR) in the cell membrane via PKC-dependent phosphorylation of the α4 subunit (Wecker et al., 2010). Furthermore, the nicotine-induced phosphorylation of PKC may be an upstream event for the increased phosphorylation of ERK1/2 (Sugano et al., 2006). Thus, the PKC-ERK signaling cascade may also contribute to nicotinic modulation of γ oscillations.

Regulation of neuronal N-methyl-D-aspartate receptors (NMDARs) by protein kinases such as PKA and PKC is critical in synaptic transmission and plasticity (Lan et al., 2001; Skeberdis et al., 2006). NMDAR activation mediates the phosphorylation of ERK and Akt/PKB (a serine and threonine protein kinase and an effector of a phosphatidylinositol 3-kinase (PI3K); Perkinton et al., 2002; Crossthwaite et al., 2004). Interestingly, NMDAR inhibition abolished the effect of nicotine on γ oscillations (Wang et al., 2015). Therefore, nicotine may modulate γ oscillations through NMDAR-mediated Ca^2+^ influx, activating MAPK.

Nicotinic modulation of synaptic plasticity is associated with the selective activation of some kinases (PKA, ERK and PI3K), but not of other kinases (tyrosine kinase, Src, Ca^2+^-calmodulin-dependent protein kinase II or p38 MAPK; Welsby et al., 2009). Therefore, in order to establish by what signaling pathways nicotine can modulate γ oscillations, we tested the role of multiple kinases, including PKA, PKC, ERK, PI3K and Akt in addition to the role of the NMDAR in nicotinic modulation of γ oscillations. We found that nicotinic modulation of γ oscillation requires both PKA and NMDAR activity and is partially blocked by PKC, ERK1/2 and PI3K inhibitors, suggesting that the NMDAR-PKA signaling pathway plays a critical role whereas PKC, ERK 1/2 and PI3K play a less critical role in acute nicotinic modulation of hippocampal γ oscillations.

## Materials and Methods

### Animals

All procedures were carried out in accordance with the UK Animals (Scientific Procedures) Act 1986 and with Animal Ethics and Administrative Council of Henan province, P.R. China, and all efforts were made to minimize animal suffering and to reduce the number of animals used. Electrophysiological studies were performed on hippocampal slices prepared from Wistar rats (male, 3–6 week-old).

Animals were anesthetized by intraperitoneal injection of Sagatal (sodium pentobarbitone, 100 mg kg^−1^, Rhône Mérieux Ltd, Harlow, UK). When all pedal reflexes were abolished, the animals were perfused intracardially with chilled (5°C), sucrose-based solution contained (in mM): 225 sucrose, 3 KCl, 1.25 NaH_2_PO_4_, 24 NaHCO_3_, 6 MgSO_4_, 0.5 CaCl_2_ and 10 glucose (305 mOsm l^−1^). Horizontal slices (450 μm) of the brain were cut in the sucrose-based solution, using a Leica VT1000S vibratome (Leica Microsystems UK, Milton Keynes, UK).

### Electrophysiological Recording, Data Acquisition and Analysis

For extracellular field potential recordings, two hippocampal slices were transferred to an interface recording chamber. The slices were maintained at a temperature of 33°C at the interface between artificial cerebrospinal fluid (ACSF) and warm humidified carbogen gas (95% O_2_–5% CO_2_). The ACSF contained (in mM): 126 NaCl, 3 KCl, 1.25 NaH_2_PO_4_, 24 NaHCO_3_, 2 MgSO_4_, 2 CaCl_2_ and 10 Glucose (pH 7.4, 310 mOsm l^−1^). The slices were allowed to equilibrate in this medium for 1 h prior to recording. The local field potential was recorded from stratum pyramidale of area CA3c, using glass microelectrodes containing ACSF (resistance 2–5 MΩ). Field potentials were amplified with an Axoprobe 1A amplifier (Axon Instruments, Union City, CA, USA) and band-pass filtered between 0.5 Hz and 2 kHz using a Neurolog NL125 (Digitimer Ltd, Welwyn Garden City, UK). Electrical interference from the mains supply was eliminated with 50 Hz noise eliminators (HumBug; Digitimer Ltd). Data were then digitized at a sample rate of 5–10 kHz using a CED 1401 plus ADC board (Cambridge Electronic Design, Cambridge, UK).

Data were analyzed off-line using Spike 2 software (Cambridge Electronic Design). Power spectra were generated using a fast Fourier transform algorithm to provide a quantitative measure of the frequency components in a 60-s recording epoch, where power, a quantitative measure of the oscillation strength, was plotted as function of frequency. The parameters used for measuring the oscillatory activity in the slice were peak frequency and γ power, calculated as the summated power in 20–60 Hz range.

All statistical tests were performed using SigmaStat software (SPSS Inc., San Francisco, CA, USA). Results are expressed as mean ± standard error of mean. Statistical significance for comparison between two groups or between three groups was determined using tests described in the text or in the figure legends, as appropriate. As γ power varies strongly between slices, the effect of drugs was expressed as a change, normalized to pre-drug values and the statistical comparisons were performed on normalized changes. Measures were considered statistically significant if *P* < 0.05.

### Drugs used for Electrophysiology

All standard reagents, except where indicated, were obtained either from Sigma-Aldrich (UK). D-(−)-2-amino-5-phosphonopentanoic acid (D-AP5), bicuculline methochloride and 2,3,-dioxo-6-nitro-1,2,3,4-tetrahydrobenzo[f]quinoxaline-7-sulfonamide (NBQX), 1,4-Diamino-2,3-dicyano-1,4-bis(o-aminophenylmercapto) butadiene (U0126), wortmannin and H-89 were purchased from Tocris Cookson Ltd (Bristol, UK). Gö6983 was purchased from Santa Cruz Biotechnology (Dallas, Tx, USA). Triciribine (TCBN) was purchased from Sigma-Aldrich (Dorset, UK). Stock solutions, at 1000 times the working concentration, were made up in water or in dimethylsulphoxide, and were stored in individual aliquots at −45°C. Working solutions were prepared freshly on the day of the experiment.

## Results

### Nicotinic Modulation of Hippocampal γ Oscillations

**Figure 1:**
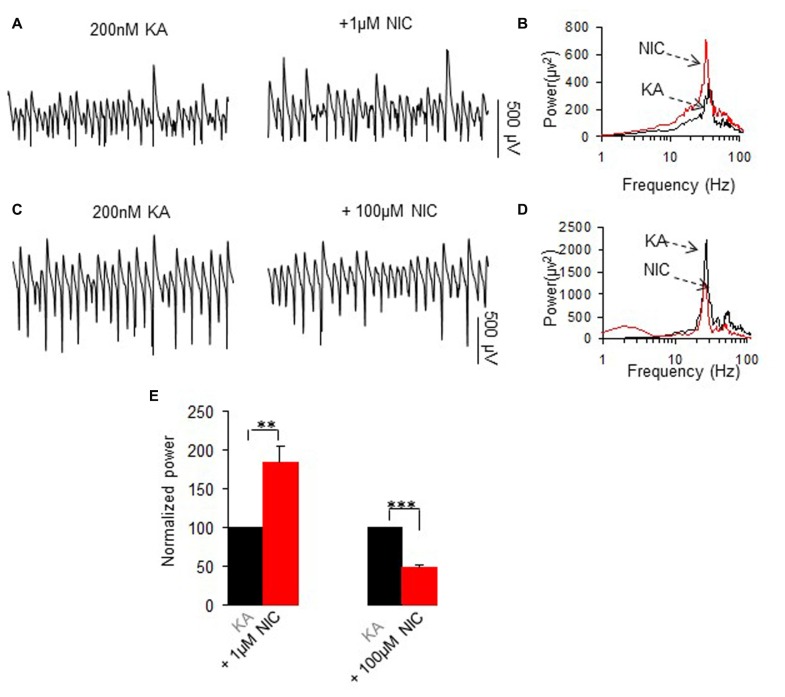
**The effects of two concentrations of nicotine on kainate-induced γ oscillations. (A,C)** Field potential recordings from the CA3 stratum pyramidale, before (the trace was taken at a time point that γ oscillation was stable for 60 min after kainate application) and after application of 1 μM nicotine (30 min after nicotine; **A**) or 100 μM nicotine (30 min after nicotine; **C**). **(B,D)** The power spectra corresponding to **(A,C)**. **(E)** γ power as % of baseline γ power for 1 μM nicotine (*n* = 13) and 100 μM nicotine (*n* = 12, **P < 0.01, ***P < 0.001, one way repeated measures (RM) ANOVA, compared with control γ power).

Oscillatory activity was induced in the slices by addition of 200 nM kainate to the ACSF and allowed to develop and stabilize for 1 h before further drugs were applied. We have previously reported that the persistent γ oscillation (20–60 Hz) induced by kainate in rat hippocampal CA3 area can be either enhanced or reduced by nicotine, depending on the concentration of nicotine used. This was confirmed in this study by acute perfusion of nicotine at 1 μM or 100 μM (Figure 1). At 1 μM nicotine caused a 83.4 ± 21.6% increase (*P* < 0.01, paired *t* test, *n* = 13) and at 100 μM a 54.9 ± 5.2% decrease (*P* < 0.001, paired *t* test, *n* = 12; Figure 1C).

Kainate-induced oscillation peak frequency was 28.4 ± 0.5 Hz, *n* = 23 and was not significantly affected by 1 μM nicotine (27.3 ± 0.8 Hz, *P* > 0.05, paired *t* test, *n* = 13) or 100 μM nicotine (26.9 ± 0.7 Hz, *P* > 0.05, paired *t* test, *n* = 12).

### The Effect of PKA Inhibition on the Nicotinic Modulation of γ Oscillations

**Figure 2:**
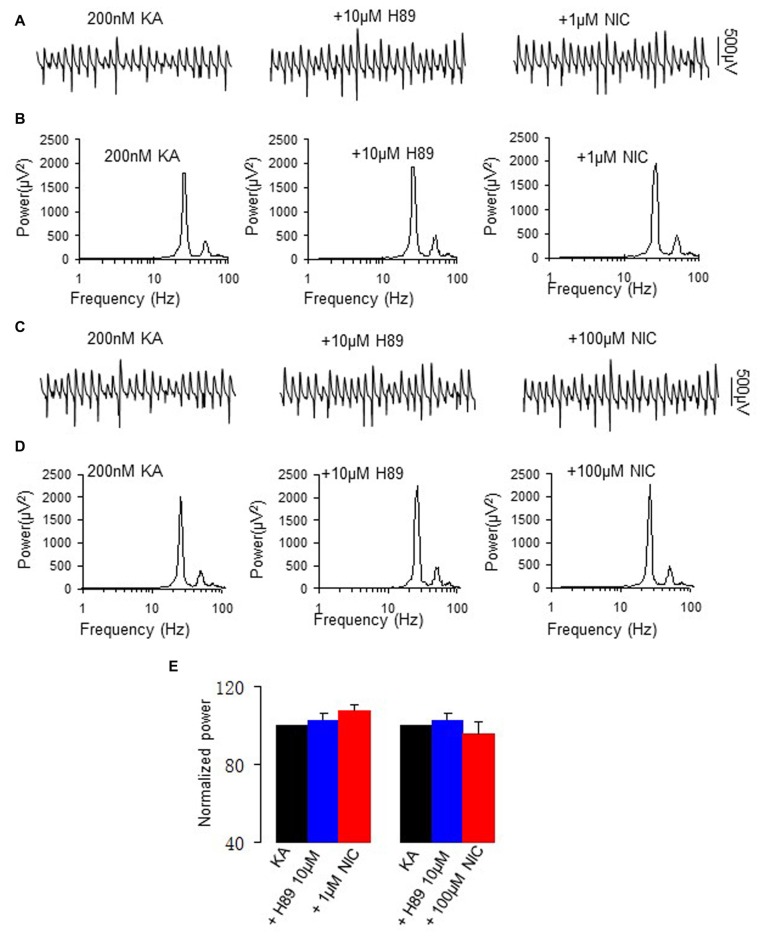
**The effect of protein kinase A (PKA) inhibitor on the nicotinic modulation of γ oscillations. (A,C)** The extracellular field potential recordings from the CA3 stratum pyramidale, for the kainate alone (control, trace taken at a time point that γ oscillation was stable for 60 min after kainate), in the presence of PKA inhibitor, 10 μM H89 (40 min after H89), and after additional application of 1 μM nicotine (30 min after nicotine; **A**) or 100 μM nicotine (30 min after nicotine; **C**). **(B,D)** The power spectra corresponding to **(A,C)**. **(E)** γ power as % of baseline γ power for control, in the presence of H89, and with additional application of 1 μM nicotine (*n* = 12) or 100 μM nicotine (*n* = 8).

To test whether PKA is involved in the modulation of γ oscillations by nicotine, we perfused the slices with the PKA inhibitor H89 (10 μM for 40 min), followed by addition of nicotine (1 μM for 30 min or 100 μM for 30 min in two different sets of experiments). H89 alone had no effect on γ power (101.6 ± 3.8% of baseline, one way repeated measures (RM) ANOVA, *P* > 0.05, *n* = 12). In the presence of H89, 1 μM nicotine caused no significant change in γ power (107.3 ± 5.6% of baseline, one way RM ANOVA, *P* > 0.05, vs. H89, *n* = 12, Figure 2). Further application of 100 μM nicotine caused no significant change in γ power neither (95.8 ± 5.9% of baseline, one way RM ANOVA, *P* > 0.05, vs. H89, *n* = 8, Figure 2). The complete block of the nicotinic modulation of γ oscillations by inhibition of PKA inhibition, suggests that PKA is required for the nicotinic modulation of γ oscillations.

### The Effect of PKC Inhibition on the Nicotinic Modulation of γ Oscillations

**Figure 3:**
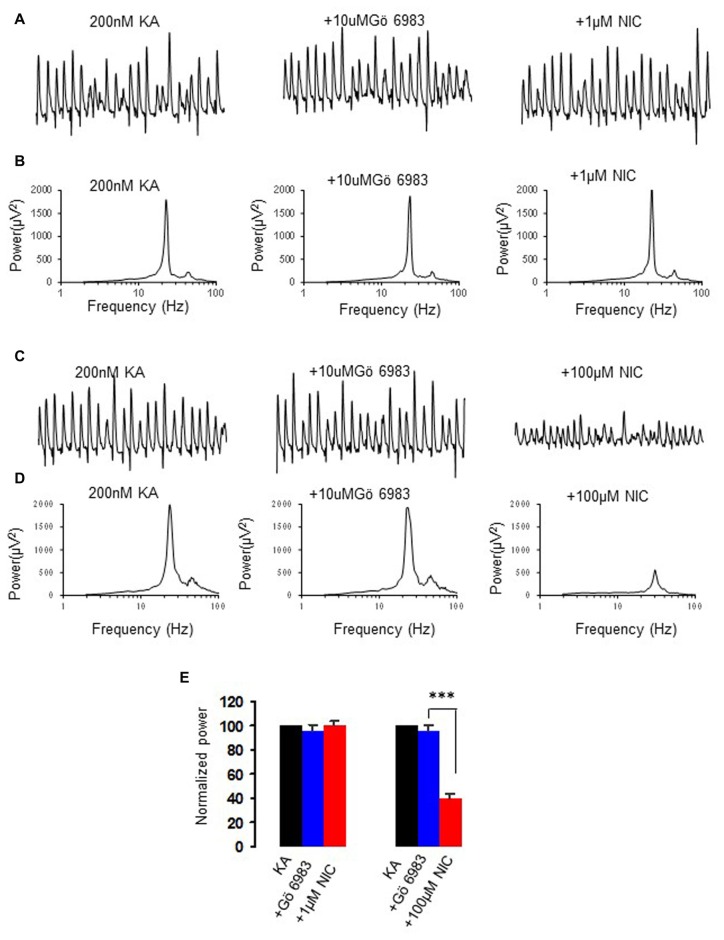
**The effect of protein kinase C (PKC) inhibitor on the nicotinic modulation of γ oscillations. (A,C)** The extracellular field recordings from CA3 pyramidale for the kainate alone, in the presence of PKC inhibitor, 10 μM Gö6983 (40 min after Gö6983), and after additional application of 1 μM nicotine (30 min after nicotine; **A**) or 100 μM nicotine (30 min after nicotine; **C**). **(B,D)** The power spectra corresponding to **(A,C)**. **(E)** γ power as % of baseline γ power for the kainate alone, Gö6983 and with additional application of 1 μM nicotine (*n* = 5) or 100 μM nicotine (*n* = 5) (***P < 0.001, one way RM ANOVA, compared with Gö6983 alone).

Nicotine activates PKC via non-α7 nAChR activation (Wecker et al., 2010). To test whether PKC is involved in the nicotinic modulation of γ oscillations, we perfused slices with the conventional PKC inhibitor Gö6983 (10 μM), followed by nicotine application. Gö6983 had no effect on γ power (95.6 ± 5.8% of baseline, one way RM ANOVA, *P* > 0.05, *n* = 5). In the presence of Gö6983 nicotine caused no significant change in γ power at 1 μM (100.6 ± 4.3% of baseline, one way RM ANOVA, *P* > 0.05, vs. Gö6983, *n* = 5, Figure 3), but caused a significant decrease in γ power at 100 μM (41.2 ± 5.6% of baseline, one way RM ANOVA, *P* < 0.001, vs. Gö6983, *n* = 5, Figure 3), which was not different from the effect of 100 μM nicotine alone (*t* test, *P* > 0.05). These results suggest that PKC is only involved in nicotinic enhancement of γ oscillations, not in the suppression at a high nicotine concentration.

### The Effect of NMDA Receptor Antagonism on the Nicotinic Modulation of γ Oscillations

**Figure 4:**
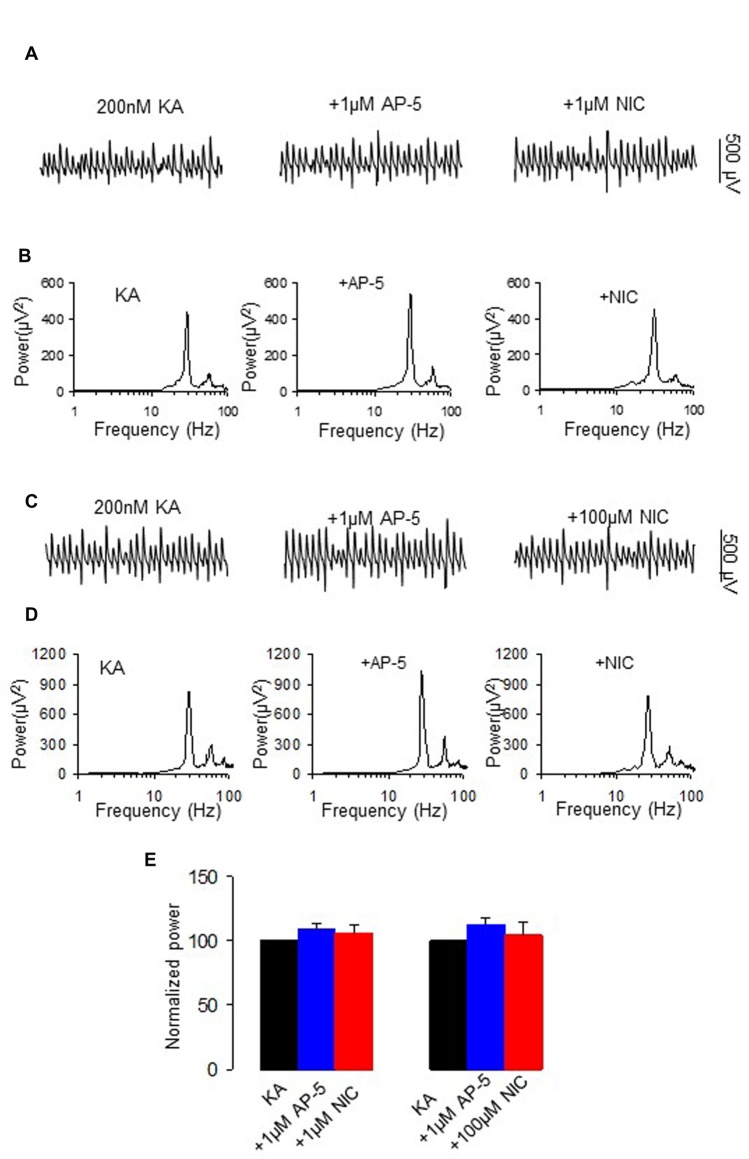
**The effect of N-methyl-D-aspartate receptor (NMDA) receptor antagonist on the nicotinic modulation of γ oscillations. (A,C)** The extracellular field recordings from CA3 pyramidale for the kainate alone, NMDA receptor antagonist, 1 μM D-AP5 (40 min after D-AP5), and after additional application of 1 μM nicotine (30 min after nicotine; **A**) or 100 μM nicotine (30 min after nicotine; **C**). **(B,D)** The power spectra corresponding to **(A,C)**. **(E)** γ power as % of baseline γ power for control, D-AP5, and with additional application of 1 μM nicotine (*n* = 8) or 100 μM nicotine (*n* = 8).

Nicotine enhances NMDA receptor function through activation of nAChR (Aramakis and Metherate, 1998; Yang et al., 2013). We further tested the role of NMDAR activation on nicotinic modulation of γ oscillations. Application of the NMDA receptor antagonist D-AP5 (1 μM) alone had no effect on γ power (109.7 ± 4.3% of baseline, one way RM ANOVA, *P* > 0.05, vs. baseline γ, *n* = 8), but blocked the nicotinic modulation of γ oscillations (example in Figures 4A,B). In the presence of D-APV, nicotine (1 μM) caused no significant change in γ power (106.3 ± 5.9% of baseline, one way RM ANOVA, *P* > 0.05, vs. D-AP5, *n* = 8, Figure 4E), which was smaller than the effect of nicotine alone (Student *t*-test, *P* < 0.001). Similarly, in a different set of experiments D-AP5 reduced the suppression of γ oscillations by 100 μM nicotine (example in Figures 4C,D), D-AP5 (1 μM) had no effect on control γ power (112.9 ± 5.3% of baseline, one way RM ANOVA, *P* > 0.05, vs. baseline γ, *n* = 8), but reduced the suppression of γ power by 100 μM nicotine (104.7 ± 10.3 of baseline, one way RM ANOVA, *P* > 0.05, vs. D-AP5, *n* = 8; Figure 4E). In comparison with the effect of 100 μM nicotine alone, the effect of D-AP5 + nicotine on γ power (11.07 ± 7.7% decrease) was significantly smaller (Student *t*-test, *P* < 0.001, vs. nicotine alone). The prevention of the nicotinic modulation of γ oscillations by D-AP5, suggests that NMDA receptor activation is required for the nicotinic modulation of γ oscillations.

### The Effect of ERK Inhibition on the Nicotinic Modulation of γ Oscillations

**Figure 5:**
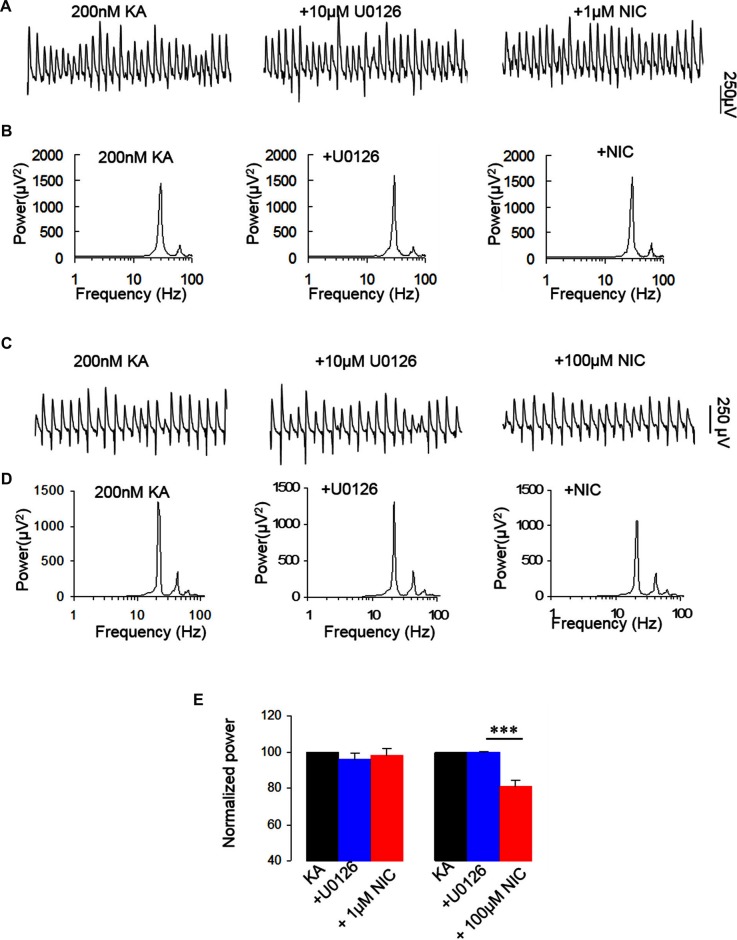
**The effect of MEK kinase inhibitor on the nicotinic modulation of γ oscillations. (A,C)** The extracellular field recordings from CA3 pyramidale for the kainate alone, in the presence of the ERK1/2 inhibitor, 10 μM 1,4-Diamino-2,3-dicyano-1,4-bis(o-aminophenylmercapto) butadiene (U0126; 40 min after nicotine), and after additional application of 1 μM nicotine (30 min after nicotine; **A**) or 100 μM nicotine (30 min after nicotine; **C**). **(B,D)** The power spectra corresponding to **(A,C)**. **(E)** γ power as % of baseline γ power for the kainate alone, U0126 and with additional application of 1 μM nicotine (*n* = 8) or 100 μM nicotine (*n* = 8; ***P < 0.001, one way RM ANOVA, compared with U0126 alone).

NMDAR activation mediates the phosphorylation of ERK (Perkinton et al., 2002), which suggests that ERK is a downstream molecule of NMDAR. ERK1 and ERK2 are two closely related MAPK. Previous studies indicate that nicotine induced phosphorylation of ERK via α7 nAChR in hippocampal neurons (Dajas-Bailador et al., 2002). We thus examined whether ERK1/2 activation is required for the nicotinic modulation of γ oscillations. U0126, a selective MEK kinase inhibitor, known to block ERK1/2 phosphorylation, was used to test the role of ERK1/2 in the nicotinic modulation of γ oscillations. U0126 (2.5 μM) alone had no effect on γ oscillations (Figure 5) and subsequent application of nicotine (1 μM) caused no significant change in γ power (101.8 ± 0.9% of baseline, *P* > 0.05 vs. U0126, *n* = 8, Figure 5). In the presence of U0126, 100 μM nicotine reduced γ power by 19.3 ± 3.0% (*P* < 0.001, vs. U0126, *n* = 8, Figure 5), which was less than the effect of 100 μM nicotine alone (49 ± 3.7% decrease, Student *t*-test, *P* < 0.001). These results suggest that ERK1/2 is involved in the nicotinic modulation of γ oscillations.

### The Effects of PI3 Kinase Inhibitor on Nicotinic Modulation of γ Oscillation

**Figure 6:**
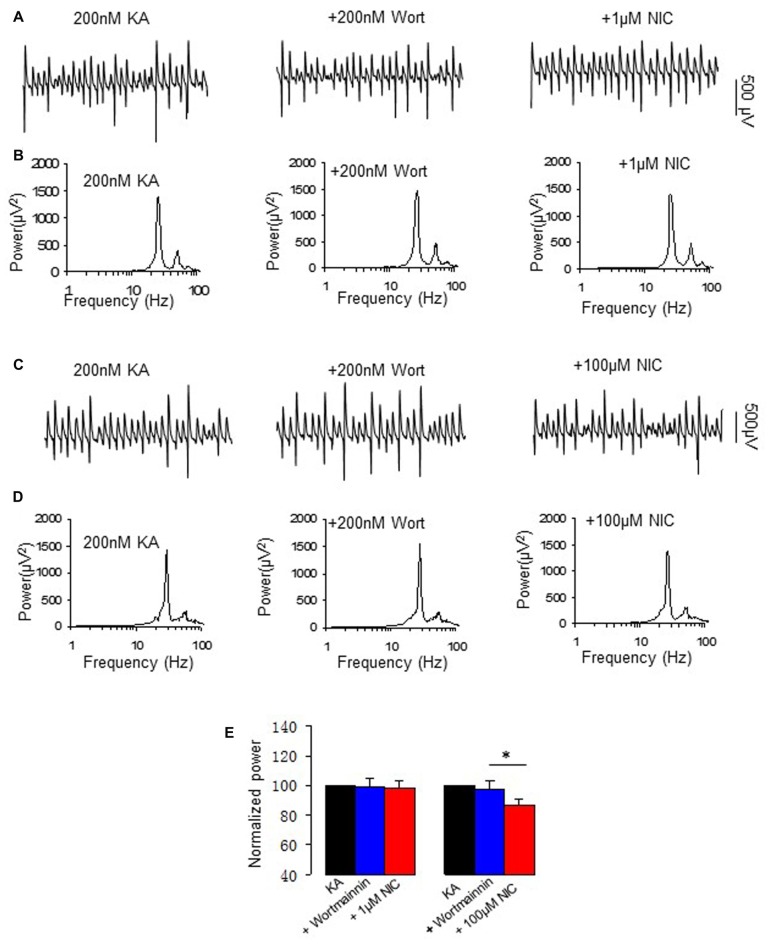
**The effect of Phosphoinositide 3-kinase (PI3K) inhibitor on the nicotinic modulation of γ oscillations. (A,C)** The extracellular field recordings from CA3 pyramidale for the kainate alone, in the presence of the PI3K inhibitor 200 nM wortmannin (40 min after nicotine), and after additional application of 1 μM nicotine (30 min after nicotine; **A**) or 100 μM nicotine (30 min after nicotine; **C**). **(B,D)** The power spectra corresponding to **(A,C)**. **(E)** γ power as % of baseline γ power for the kainate alone, wortmannin, and with additional application of 1 μM nicotine (*n* = 8) or 100 μM nicotine (*n* = 8; *P < 0.05, one way RM ANOVA, compared with wortmannin alone).

PI3K is a central mediator of NMDA receptor signaling to Erk1/2 (Perkinton et al., 2002) and α7 nAChR activation reduced amyloid β neurotoxicity through PI3K activation (Kihara et al., 2001). We therefore tested whether PI3K is involved in the effect of nicotine on γ oscillations, using wortmannin, a cell-permeable, irreversible inhibitor of PI3K. Wortmannin (0.2 μM) alone had no effects on γ oscillations (104.5 ± 9.3% of baseline, *P* > 0.05, *n* = 8). Subsequent application of nicotine (1 μM) caused no significant change in γ power (102.1 ± 5.3% of baseline, *P* > 0.05, vs. wortmannin, *n* = 8). In the presence of wortmannin the 100 μM nicotine-induced reduction of γ was 13.2 ± 2.8% (*P* < 0.05, vs. wortmannin, *n* = 8, Figure 6), which was smaller than that of 100 μM nicotine alone (49 ± 3.7% decrease, Student *t*-test, *P* < 0.01). These results suggest that PI3K is involved in the nicotinic modulation of γ oscillations.

### The Effects of Akt Inhibitor on Nicotinic Modulation of γ Oscillation

**Figure 7:**
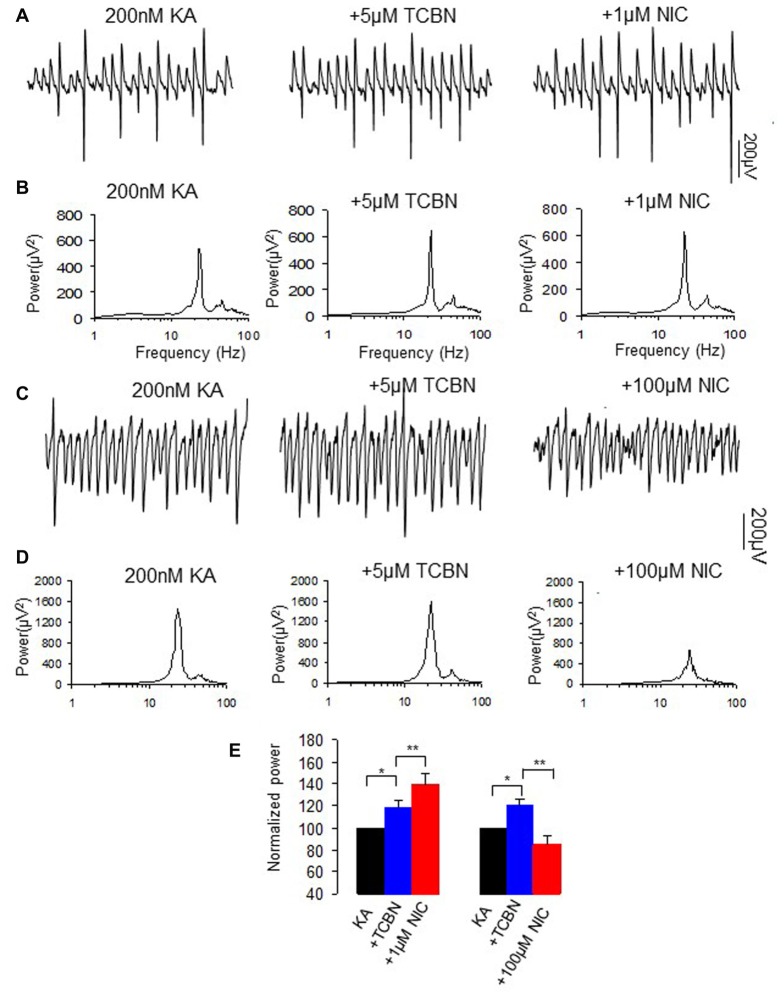
**The effects of Akt inhibitor on the nicotinic modulation of γ oscillations. (A,C)** The extracellular field recordings from CA3 pyramidale for the kainate alone, in the presence of Akt inhibitor 5 μM TCBN (40 min after nicotine), and after additional application of 1 μM nicotine (30 min after nicotine; **A**) or 100 μM nicotine (30 min after nicotine; **C**). **(B,D)** The power spectra corresponding to **(A,C)**. **(E)** γ power as % of baseline γ power for the kainate alone, TCBN, and with additional application of 1 μM nicotine (*n* = 9; *P < 0.05, **P < 0.01, one way RM ANOVA, compared with TCBN alone) or 100 μM nicotine (*n* = 9; *P < 0.05, **P < 0.01, one way RM ANOVA, compared with TCBN alone).

NMDAR activation also mediates the phosphorylation of Akt/PKB in addition to that of ERK (Perkinton et al., 2002). We then tested whether Akt, a serine and threonine protein kinase and an effector of a PI3K, affects nicotinic modulation of γ oscillations, using Triciribine (TCBN), a selective Akt inhibitor. TCBN (5 μM) caused a small but significant increase in γ power (118.1 ± 6.5% of baseline, *P* < 0.05 vs. baseline, *n* = 9). Subsequent application of nicotine (1 μM) caused a 23.3 ± 11.3% increase in γ power (*P* < 0.01, vs. TCBN, *n* = 9, Figure 7), which is smaller than that in the absence of TCBN (Student *t*-test, *P* < 0.05). In the presence of TCBN, 100 μM nicotine caused a 36.7 ± 7.7% decrease in γ power (*P* < 0.01, vs. TCBN, *n* = 9, Figure 7), which was not different from the effect of nicotine alone (Student *t* test, *P* > 0.05). These results indicate that Akt activation is involved in the nicotinic modulation of γ oscillations.

## Discussion

In this study, we found that activation of a PKA/PKC-NMDAR-ERK/PI3K signaling pathway is required for nicotinic enhancement of γ oscillations, with a partial involvement of Akt. PKA and NMDAR activation are also required for inhibition of γ oscillations by high concentration of nicotine, with a partial involvement of the downstream signaling kinase ERK or PI3K. None of above-mentioned kinases except for Akt is directly involved in the generation of γ oscillations.

### Signaling Pathways Potentially Involved in the γ Oscillation Enhancing Effect of Low Concentrations of Nicotine

Our data are consistent with the nicotinic enhancement of auditory evoked γ oscillations in mouse hippocampus via α4β2 nAChR activation at low micromolar concentrations (Phillips et al., 2007; Featherstone et al., 2012). The nicotinic γ oscillation enhancement may be related to the activation of NMDAR and subsequent calcium influx, as D-AP5, in line with previous observations (Wang et al., 2015), completely blocked the effects of nicotine. Calcium influx through the NMDAR is the start of many signaling pathways that alter neuronal and network functions (Perkel et al., 1993; Sobczyk and Svoboda, 2007). The lack of effect of the NMDAR antagonist on γ oscillations replicates our previous findings (Wang et al., 2015; Chen et al., 2016) and that of others (Cunningham et al., 2003; Fisahn et al., 2004). It indicates that under normal slice conditions there is little NMDAR activation. How can NMDAR inhibition have such an effect on the nicotinic enhancement of γ oscillations? One explanation is that nAChR receptor activation causes an enhancement of NMDAR activation to levels that give substantial calcium influx and calcium-dependent signaling pathways that subsequently affect properties that determine γ oscillation strength. Indeed, it has been reported that α7 nAChRs form a protein complex with the NMDAR through a protein-protein interaction, and activation of α7 nAChR upregulates NMDAR-mediated whole cell currents (Li et al., 2013). With both PI3K and ERK1/2 activity required for the γ oscillation-enhancing effect of nicotine, one potential downstream signaling pathway has been identified. ERK1/2 is known to be one of the downstream molecules of NMDAR activation and PI3K is a central mediator of NMDAR signaling to ERK1/2 (Perkinton et al., 2002; Crossthwaite et al., 2004; Skeberdis et al., 2006).

**Figure 8:**
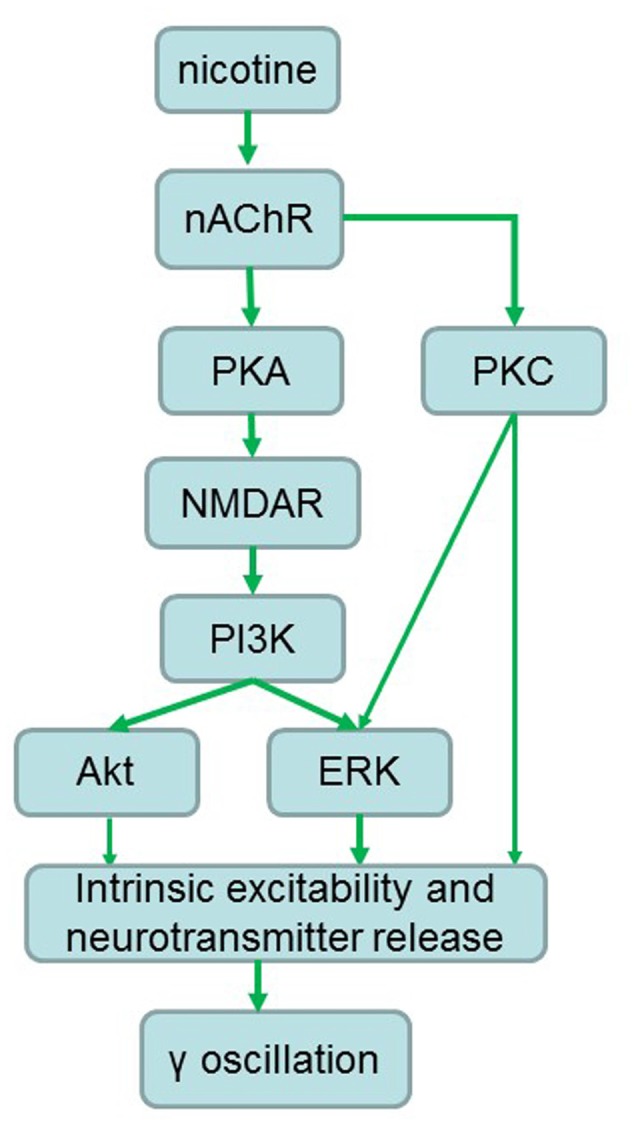
**Proposed signaling pathways involved in the nicotinic enhancement of gamma oscillations.** Green arrows indicate faciliatory relationships.

Stimulation of α7 nAChR activates PI3K in cultured cortical neurons (Kihara et al., 2001) and activation of nAChRs engages calcium-dependent signaling pathways including PI3K (Liu et al., 2008). The ERK activation may be a common pathway for both nAChR and NMDA receptor activation, as stimulation of α7-nAChRs activates calcium-dependent intracellular signaling molecules including ERK1/2 (Chang and Berg, 2001; Hu et al., 2002; Gubbins et al., 2010). Inhibition of PKA blocked the nicotine-induced ERK1/2 activity in hippocampal neurons in cultures (Dajas-Bailador et al., 2002), thus PKA could be an upstream kinase of ERK1/2 (Figure 8). Although our study is not exhaustive we have identified the PI3K-ERK1/2 pathway as essential for the NMDAR-mediated nicotinic enhancement of γ oscillations. Akt is a putative downstream molecule of PI3K and a downstream molecule of NMDAR activation, since it was reported that in striatal neurons NMDAR-dependent Akt activation was mediated by PI3K (Crossthwaite et al., 2004). Akt was only partially involved in the γ oscillation enhancement. The Akt inhibitor caused an increase in γ oscillations suggesting that Akt activation is suppressing γ oscillations *in vitro*, which is in agreement with our recent observation (Wang et al., 2016).

How can NMDAR-mediated activation of the PI3K-ERK/Akt signaling pathway affect γ oscillation strength? γ oscillations emerge from the rhythmic IPSCs generated by interneurons, activated by pyramidal cells in a feedback manner that, in turn, synchronize the firing of neurons (Mann and Paulsen, 2005). If acting on interneurons the PI3K-ERK/Akt pathway could affect γ oscillations by enhancing intrinsic excitability and GABA release. Although the signaling pathway involved is unknown, α7 nAChR activation modulates hippocampal interneurons and enhances GABA release (Hulo and Muller, 2001; Wanaverbecq et al., 2007), which would increase γ oscillations. If acting on pyramidal neurons the PI3K-ERK/Akt pathway could affect γ oscillations by increasing intrinsic excitability and glutamate release.

PKA activation was also required for the nicotinic enhancement of γ oscillations and can form the link between nAChR activation and NMDAR-mediated signaling pathways responsible for the enhancement. PKA can be activated by nAChR activation (Dajas-Bailador et al., 2002) and can, in turn, phosphorylate NMDARs, causing an increase in Ca^2+^ permeability (Skeberdis et al., 2006). It is, however, remarkable that this NMDAR-mediated calcium influx is required while nAChR are Ca^2+^ permeable themselves (Didier et al., 1995). Previously we have shown that for the full nicotinic γ oscillation enhancement both α_4_β_2_ and α_7_ type nAChRs are required, but mostly the α_4_β_2_ type (Wang et al., 2015), which is less Ca^2+^ permeable than the α_7_ type (Fucile, 2004). Furthermore, the location of the limited calcium influx through nAChRs may be different from the location of the NMDAR-mediated calcium influx required to activate the signaling pathway responsible for the modulation of γ oscillations.

PKC activation was also required for the nicotinic enhancement of γ oscillations. PKC activation can increase NMDA channel opening and insert more NMDA channels into the plasma membrane (Lan et al., 2001), thus increasing NMDAR-mediated calcium influx. However, since both PKA and PKC activation is required, it is unlikely that PKC and PKA activation converge on NMDAR modulation, because then the effects would be additive. PKC activates ERK1/2 (Cox et al., 2004), so could also converge with the PKA-NMDAR-PI3K pathway to activate ERK1/2 in a way that requires both. Alternatively, it is possible that PKC activation act directly on one or more of the γ oscillation strength determining factors (intrinsic excitability and neurotransmitter release), in co-dependence with the PKA-NMDA-IP3K-ERK/Akt pathway (Figure 8).

Impaired γ oscillations have been associated with cognitive deficits typical to schizophrenia (Cho et al., 2006) and Alzheimer’s disease (Klein et al., 2016). Nicotine use is increased in schizophrenia patients, where it has cognitive benefits (Boggs et al., 2014). Also nicotine increases visual attention and working memory in healthy volunteers (Ernst et al., 2001). It is possible that this is due to nicotinic enhancement of γ oscillations, which was significant at concentrations as low as 0.1 μM (Wang et al., 2015), comparable to plasma levels achieved by cigarette smoking. Further elucidating the pathways involved may well lead to identifying therapeutic targets that can increase γ oscillations effectively and safely.

A limitation of this study is that we have not defined the specific cell subtypes that are involved in the nicotinic enhancement of γ oscillations. It appears that expression of nAChR subtypes in hippocampus is highly selective, which determines the precise modulation of specific synapse function (Leão et al., 2012). Thus, defining the specific cell type which may contribute to the nicotinic enhancement of γ oscillations has to be to the focus of future studies.

### Signaling Pathways Potentially Involved in the γ Oscillation Suppressing Effect of High Concentrations of Nicotine

Both blocking NMDARs and PKA inhibition completely prevented the suppression of γ oscillations at a high concentration of nicotine, whereas the downstream molecules in the pathway (ERK, PI3K) only partially contributed to the suppression, and PKC and Akt were not involved. These results suggest that nicotinic enhancement or inhibition of γ oscillations involves both shared and different mechanisms.

The suppressing effect of high concentrations of nicotine was stronger in the presence of nAChR antagonists (Wang et al., 2015), which seems to indicate an effect of nicotine on the NMDAR bypassing the nAChR. One possibility is that the intensity of NMDAR activation and subsequent calcium influx may differentiate between enhancement (low calcium influx) and suppression (high calcium influx). Because γ oscillations are a high energy demanding neuronal activity (Kann et al., 2011), a large calcium influx may overwhelmingly overcome the role of downstream kinases such as ERK, PI3K or Akt, causing the collapse in γ oscillations via mitochondrial membrane depolarization (Kann et al., 2011; Lu et al., 2012). A reverse correlation between intracellular calcium and γ oscillations was observed in aged hippocampal neurons (Lu et al., 2011, 2012). The physiological role of the γ oscillation-suppressing effect of high concentrations of nicotine is limited, because plasma nicotine levels will normally not exceed 1 μM.

### Possible Cellular Mechanisms of Nicotinic Modulations of γ Oscillations

Our results reveal that activation of a signaling pathway of PKA/PKC-NMDA-ERk/PI3K contributes to the nicotinic modulation of γ oscillations. Nicotine may increase NMDAR activation through PKA- and PKC-dependent phosphorylation, which leads to the Ca^2+^ influx and activation of the downstream kinases such as ERK, PI3K and Akt and subsequently modulation of γ oscillations. The nicotinic modulation of γ oscillations may depend on the intensity of NMDAR activation, a weak or strong activation of NMDAR by a low or high concentration of nicotine enhances or inhibits γ, respectively (Figure 8).

## Author Contributions

CL designed the experiment; JW, FG, XH, XC, YW and XW performed the experiments; CL, JW, ZF, XH and MV wrote the manuscript; CL, JW, XH, XC, YW and XW analyzed the data. All authors reviewed the manuscript.

## Conflict of Interest Statement

The authors declare that the research was conducted in the absence of any commercial or financial relationships that could be construed as a potential conflict of interest.
